# Impact of Methods on the Measurement of mRNA Turnover

**DOI:** 10.3390/ijms18122723

**Published:** 2017-12-15

**Authors:** Takeo Wada, Attila Becskei

**Affiliations:** Biozentrum, University of Basel, Klingelbergstrasse 50/70, 4056 Basel, Switzerland; takeo.wada@unibas.ch

**Keywords:** posttranscriptional regulation, *Saccharomyces cerevisiae*, nonsense mediated decay, NMD, splicing, 4-thiouracil, 4sU, *rpb1-1*, exponential decay

## Abstract

The turnover of the RNA molecules is determined by the rates of transcription and RNA degradation. Several methods have been developed to study RNA turnover since the beginnings of molecular biology. Here we summarize the main methods to measure RNA half-life: transcription inhibition, gene control, and metabolic labelling. These methods were used to detect the cellular activity of the mRNAs degradation machinery, including the exo-ribonuclease Xrn1 and the exosome. On the other hand, the study of the differential stability of mature RNAs has been hampered by the fact that different methods have often yielded inconsistent results. Recent advances in the systematic comparison of different method variants in yeast have permitted the identification of the least invasive methodologies that reflect half-lives the most faithfully, which is expected to open the way for a consistent quantitative analysis of the determinants of mRNA stability.

## 1. Introduction

mRNA turnover is determined by the rates of mRNA synthesis and degradation, which jointly adjust the level of gene expression [1,2,3].

The degradation of mRNAs in eukaryotes occurs largely in the cytoplasm and begins with the removal of the poly(A) tail. Subsequently, the degradation can proceed through two major pathways [4]. The mRNAs are processed either by the Xrn1p-mediated 5′ to 3′ degradation pathway after decapping or by the exosome (3′ to 5′) without decapping [5]. The molecular mechanisms have been already extensively reviewed [5].

Despite the central role of these enzymes in mRNA degradation, mutations in them often result in specific pathologies. For example, mutations in the RNA exosome cause neurodegenerative diseases [6]. Sequence variations in *XRN1* have been frequently associated with susceptibility to bacterial diseases, such as *Staphylococcus aureus* infections, and have been also implicated in the altered defense against viruses [7,8].

**Figure 1:**
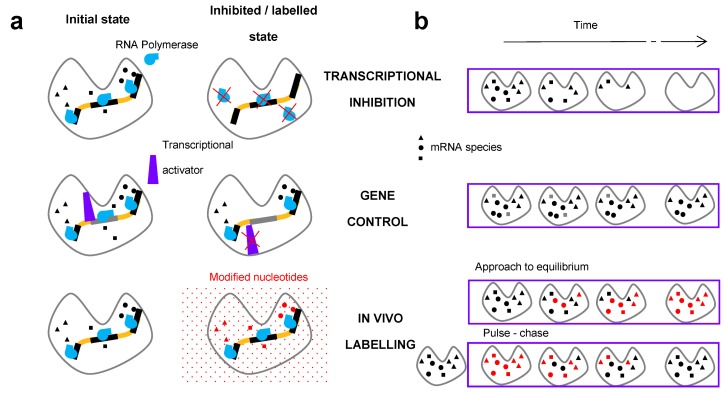
Main classes of methods to study RNA stability. (**a**) Scheme of the molecular mechanism affected by the specific method procedures. In transcriptional inhibition, the RNA polymerase is inactivated; the expression of all genes is reduced. In the gene control method, a transcriptional activator dissociates from a specific promoter, shutting off the expression of the specific gene under the control of this promoter. For labelling of the RNAs, modified nucleotides are introduced into the cell (red dots), which are then incorporated into the RNA; (**b**) Time course of the experiments to determine RNA half-lives. Inhibition of transcription of the gene(s) is triggered at *t* = 0 in transcriptional inhibition and gene control methods. There are two subclasses of the in vivo labelling. In the approach to equilibrium method, a pulse of modified nucleotides is applied and the increase of the labelled mRNA is monitored. In the pulse–chase method, the RNA is first labeled (pulse period). During the chase period starting at *t* = 0, the labeled nucleotides are washed out and replaced with unlabeled nucleotides and the decline of the labelled RNA is monitored.

mRNA half-lives have been measured since the inceptions of molecular biology [1,2,3]. Three main classes of methods have been available to study mRNA degradation rates: transcriptional inhibition, gene control, and metabolic in vivo labelling [9] (Figure 1). Transcriptional inhibition and in vivo labelling have been intensively used for the genome-wide measurement of mRNA half-lives for more than four decades. Despite the long history of mRNA half-life measurements, recent studies have revealed that different methods used to measure mRNA half-life often yield inconsistent data [10,11,12].

We will discuss the advantages and critical points of the employed methods and how they can be compared and optimized. We will also review the identification of the main components of the degradation machinery from a historical-methodological perspective. Since these pathways were identified in the budding yeast *Saccharomyces cerevisiae*, we will focus on this eukaryotic model organism.

## 2. Methods for the Measurement of RNA Degradation Rates

### 2.1. In Vivo Metabolic Labelling

**Figure 2:**
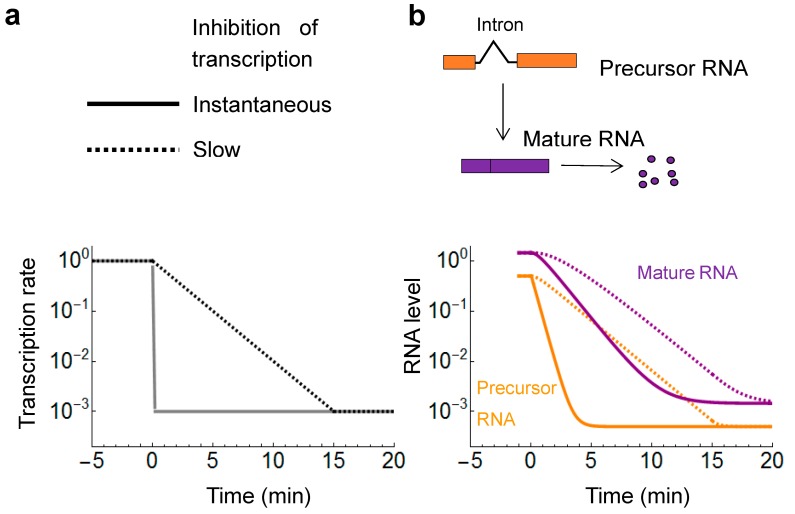
Impact of the speed of transcriptional inhibition on the measured mRNA half-lives. (**a**) Two different scenarios are shown for the inhibition of transcription. In the ideal case, the inhibition occurs instantaneously down to a baseline level (full line). In suboptimal cases, inhibition of transcription ensues with slow kinetics (dashed line); (**b**) The decay of RNA upon instantaneous (full lines) and slow (dashed lines) inhibition of transcription. The precursor mRNA (orange), containing the intron, can be used to monitor the inhibition kinetics since the precursor is converted rapidly to mature mRNA (purple) by splicing. The half-life of the mature mRNA (purple) will appear much longer with the slow inhibition kinetics and there is also a longer lag period before the mRNA level starts to decline.

Common to the variants of this method is that modified nucleotides are introduced into the cells to label the mRNA (Figure 2a, bottom panel). The RNA half-life can be deduced by quantifying the rate at which the labelled RNA increases or declines after the introduction or removal of the labelled nucleotides, respectively (Figure 2b, bottom panel). The labelling chemistry has changed over the five decades of the method’s employment. Initially, radioactively labelled nucleotides were used, especially [^3^H]-adenine and [^32^P]-phosphate [1,3]. Thereafter, their use has declined for several reasons. First, radioactivity may elicit cellular damage, including DNA double-strand breaks [13], and may trigger cellular signaling that alters transcription and RNA stability. Second, the invention of qPCR and high-throughput RNA detection technologies paved the way for the spread of labelling with non-radioactively modified nucleotides so that the labelled RNA can be separated from the total RNA to be quantified. If the RNA contains bromouracil (BrU), the separation is performed by immunoprecipitation. If the RNA incorporates 4-thiouracil, it can be biotinylated followed by binding to streptavidin beads [14,15]. The biotinylation-based separation has prevailed recently.

Interestingly, the uptake of 4-thiouracil and 4-thiouridine differs in mammalian and yeast cells. Uridine is a nucleoside containing uracil attached to a ribose. In yeast, only uracil and not uridine is imported into the cell efficiently. The intracellular uracil is then converted by the pyrimidine salvage pathway enzyme, the uracil phosphoribosyltransferase (Fur1) to uridine monophosphate, UMP [16]. In contrast, both compounds are transported into a mammalian cell but only uridine is incorporated into the RNA because the above salvage pathway is inactive [17]. For this reason, 4-thiouridine is mostly used in mammalian cells [14,15] and 4-thiouracil is used in yeast cells [18]. Initially, 4-thiouridine was also used in yeast cells. To enable the uptake of 4-thiouridine (4sU), the human equilibriative nucleoside transporter (hENT1) was expressed in yeast cells [19]. Subsequently, 4-thiouracil (4TU) was shown to be efficiently incorporated into yeast RNA [18], which permitted the use of a simpler chemical without the need to express exogenous genes in yeast cells.

Each nucleobase derivative has different impact on cell physiology. Bromouridine is less toxic than 4-thiouridine in mammalian cells [20]. At elevated concentrations of 4-thiouridine (>50 µM), which are usually used for mRNA labeling experiments, the production and processing of rRNA is inhibited [21]. Thus, in vivo labeling can trigger a nucleolar stress response, which can interfere with the RNA stability measurements.

The RNA half-lives can be determined by pulse–chase or by approach to equilibrium (Box 1). When the approach to equilibrium is followed, the increase of the labelled RNA upon addition of the modified nucleotides (pulse) is monitored. The rate of increase in the labelled RNA depends on the degradation rate [2], and not on the synthesis rate. In the pulse–chase method, a pulse of labelled nucleotide is added to the cells. In the subsequent chase period, the cells are washed with media containing unlabeled nucleotides and the decline of the labelled RNA is monitored, as in the classical decay experiments.

It is important to note that by varying the duration of the pulse, one can focus on different time-scales of the RNA turnover [22]. Short pulses are particularly suitable to study fast processes, such as RNA splicing.

### 2.2. Transcriptional Inhibitors

When RNA expression is inhibited, all RNA species start to decay and by quantifying their change over time, their half-lives can be determined. In the earliest experiments, only cytoplasmic RNA expression was inhibited by blocking the export of RNAs into the cytoplasm; subsequently, transcription was inhibited to block the expression of RNAs completely. The inhibition of RNA expression can be achieved by small-molecule inhibitors or by creating temperature-sensitive alleles. The *rna1* was one of the earliest examples of temperature-sensitive alleles that was used to determine RNA half-lives [23]. The *RNA1* gene encodes a RanGAP, which generates the nucleocytoplasmic RanGTP gradient to drive the nucleocytoplasmic transport. Its inactivation causes a collapse of the gradient and transport [24]. By inhibiting RNA export, the level of cytoplasmic RNA and polyribosomes declines. Thus, the polyribosome fraction or the instantly synthesized proteins can be measured to infer the amount of cytoplasmic mRNAs [25]. Later, inhibition of the polymerase became the most widely used technique, which was facilitated by the isolation of the *rpb1-1* allele. The standard name of *RPB1* in budding yeast is *RPO21* and it encodes the largest subunit of the RNA polymerase II [26].

Box 1 **Fitting of parameters to determine RNA half-lives.**

Just like radioactive decay, the decay of mRNA molecules is typically described by an exponential function.

(1) $$R\left(t\right)=R_{0}e^{-kt}$$

*R*_0_ and *R*(*t*) denote the RNA level at the initial and subsequent time points, respectively, and the decayrate constant is *k* = Ln [2]/*t*_1/2_.

When the initial level of the RNA is zero then the time to reach the steady-state (equilibrium) is determined also by the decay rate, and not—as often incorrectly assumed—by the synthesis rate, *p*.

(2) $$R\left(t\right)=\frac{p}{k}\left(1-e^{-kt}\right)$$

The above equation and the half-life in it can be fitted to the time series of (labelled) RNA levels upon induction of gene expression or upon addition of modified nucleotides. The time to reach the half-saturation corresponds exactly to the half-life [1,2,3].

Equation (1) is used to fit half-lives in transcriptional inhibition and gene control experiments, while both Equations (1) and (2) are used for in vivo labelling experiments.

Transcriptional inhibition can be used for genome-wide measurements of RNA decay, which contributed to the popularity of this approach, even though potential disadvantages have been known. The stepwise increase of temperature required for the thermal inactivation of the polymerase and the rapid loss of labile factors upon transcriptional inhibition may have pleiotropic effects on cell physiology. A study using metabolic labelling has revealed that the *rpb1-1* allele alters the mRNA stability even at permissive temperature [11].

Chemicals, such as 1,10-phenanthroline and thiolutin, have been also used to inhibit the RNA polymerases. They, too, have their disadvantages. 1,10-phenanthroline, a heterocyclic compound, inhibits a large number of enzymes in addition to the RNA polymerase, especially zinc metalloproteases [27]. The action of thiolutin, a dithiolopyrrolon antibiotic isolated from the *Streptomycetes luteosporeus*, has been thought to be a more specific inhibitor of transcription. Interestingly, recent studies have unveiled that thiolutin can also chelate Zn, and inhibit metalloproteases, including a deubiquitinating enzyme of the proteasome [28,29]. Furthermore, the mRNA half-life measured with thiolutin strongly depends on the applied concentrations [30].

To inhibit transcription in mammalian cells, mostly actinomycin D has been used, which is an antibiotic isolated from the *Streptomyces parvulus*. It inhibits the elongating RNA polymerase (RNAP). Elongation blockage induces the proteasomal degradation of RPB1, mediated by the p53 tumor suppressor [31]. RPB1 is the subunit 1 of the RNA polymerase II. While actinomycin D inhibits all isoforms of the RNA polymerases, α-amanitin is highly selective for RNAP II and RNAP III but its action is slow. New compounds have also been identified that are fast, selective and able to completely arrest transcription by triggering rapid degradation of RNAP II [32], which may reduce the side-effects of transcriptional inhibition to study mRNA turnover.

### 2.3. Gene Control

Gene control and transcriptional inhibition are related methods since gene expression is shut off, and the mRNA level starts to decline. The two methods differ with respect to the scale of inhibition. With transcriptional inhibition, the expression of all genes is inhibited. On the other hand, the expression of a single gene is shut off in the gene control method, in which a gene is placed under the control of a regulatable promoter, such as the GAL or the TET promoter [9]. Despite the lesser probability of having side effect, the gene specific control has been rarely used because each experiment only yields the half-life of a single mRNA.

The advantage of the TET system is that it is of bacterial origin and thus orthogonal to the endogenous processes in eukaryotes. Doxycycline, which is often used to control the TET system, has no or minimal effect on the expression of the *S. cerevisiae* genome [33]*.* Doxycycline dissociates the tetracycline transactivator (tTA) from the promoter. It is important not to use a too strong promoter to express the tTA because high expression of the activator may cause cellular growth defects and major alterations in gene expression [34]. A moderately strong promoter (e.g., *MYO2)* generates sufficient expression of tTA without causing growth defects [35].

Alternatively, a TetR-repressor fusion protein can be recruited to *tet* operators inserted into promoters to shut off gene expression, using classical general repressors such as Ssn6p or Sum1p [12]. This strategy has the advantage that it is even less invasive since the original promoter sequence is retained upon the insertion. On the other hand, not all repressors have a fast action and not all promoters can be efficiently repressed.

The TET system can be used also in mammalian cells. Pre-exposure of cells to low concentration of doxycycline was shown to improve the decay kinetics, possibly because the reduced expression of some genes eliminated the side effects [36]. Thus, the system may require prior optimization. Since most mammalian genes are very long and contain multiple introns, the cloning of mammalian genes into plasmids is limited technically, which can be a serious impediment to implement the gene control method. The cloning of the full length gene is desirable as it has been known that mRNA processing, including splicing, is strongly dependent on the chromatin state [37].

The *GAL* promoters in yeast are active in the presence of galactose and are repressed by glucose. When cells are grown in glycerol or in the neutral sugar raffinose, the *GAL* promoters are inactive. The expression of most genes in the inactive state is similar to or slightly higher than the expression in the repressed state [38]. To shut off *GAL* driven transcription in cells grown in galactose, glucose is added at high concentrations [3]. It is important to note that glucose triggers a signal that can transiently increase the decay rate of some mRNAs [38]. Therefore, the decay rate may not reflect the steady-state turnover for these mRNAs. To avoid the transient signaling due to the shift from galactose to glucose, galactose can be washed out and replaced by the neutral sugar, raffinose [12], which results in half-lives similar to those obtained with the TET system [38]. In this case, it is recommended to use lower galactose concentration for induction to expedite its transport out of the cell [39]. In fact, the high galactose concentrations (2%), used in most studies, are not needed because the *GAL* genes can be already induced at as low concentration as 0.05% [38].

### 2.4. Additional Methods

The above three major classes of methods yield RNA half-lives directly. There are also methods that can be used to estimate half-lives indirectly. For example, the half-life can be calculated when the RNA synthesis rate constant and the mRNA concentration are known. The mRNA synthesis rates can be measured by genomic run-on experiments by stopping transcription and by resuming it in the presence of labelled nucleotides so that the nascent transcripts are extended [40].

In principle, it is also possible to deduce mRNA half-lives from steady-state expression of RNAs measured in single cells, provided the regulating transcription factor undergoes large-amplitude nucleocytoplasmic oscillations. [41]. Upon export of a transcription factor to the cytoplasm, the decline of mRNA can be observed in single cells and the RNA half-life can be in principle estimated. Such singe cell observations require the insertion of stem-loops into the RNA. Since stem-loops can affect RNA processing [42], the mRNA stability has to be assessed before and after insertion of stem-loops.

## 3. Comparison of the Average mRNA Half-Lives

Ever since RNA stability was studied, it has been assumed that each method can affect the measured half-lives. Consequently, mRNA half-lives obtained by different methods have been repeatedly compared. Two measures have been used for the comparison: the average half-life and the correlation.

There are two common measures of the average: the mean and the median. The mean half-life has been mostly reported in the earlier studies, while the median is mostly reported in the later ones. The median is typically larger than the mean because the distribution of the half-lives is often skewed to longer half-lives. The overwhelming majority of the studies yielded average mRNA half-lives between 10 and 25 min, using in vivo labelling or transcriptional inhibition [12,25,43]. A recent study reported a median half-life of around 2 min, representing a substantial deviation from the above range [12]. Such short-lives have been typically found only in prokaryotes [44].

What could be the reason for the considerable differences among the measured half-lives? In an ideal case, the inhibition transcription or the incorporation of labelled nucleotides occurs instantaneously after starting the experiment. In practice, these processes occur after a lag period or with a slower than expected kinetics. Using dual labelling experiments, the lag time due to the incorporation of nucleotides has been estimated to be around 5 min in yeast [1].

Inhibition of the polymerase can also introduce a lag that is dependent on the gene length because initiation and elongation is inhibited with different efficiencies [44].

A simple exponential model of decay assumes that the inhibition of transcription is instantaneous or occurs very rapidly (Box 1). This may or may not be true and should be verified. It is not easy to monitor directly how rapidly transcription declines. The level of the un-spliced precursor mRNA provides a good proxy for the precipitously changing transcription rate because splicing of the precursor mRNA is typically faster than the RNA decay (Figure 2). To monitor transcription, mRNAs have to be selected that undergo a fast, efficient splicing, which can be inferred from the ratio of the mature to the precursor RNA. The larger the mature-to-precursor ratio, the faster the splicing. If the ratio is 100 then the precursor amount is expected to decline 100 times faster via splicing than the mature mRNA via decay. At lower splicing rates, the formula for this ratio has a correction term that takes into account the escape of the unspliced precursor mRNA from the nucleus to the cytoplasm [45,46].

Despite the importance of this control experiment, relatively few studies have reported the half-time of the precursor RNA [47,48]. The reported values range between 0.5 and 1 min, which constitute the lower limit of the half-lives that can be reliably detected. If the experimental set-up is not optimal and the precursor declines slowly, the experimental conditions can be modified to accelerate the transcriptional inhibition. For example, thermal inactivation of the polymerase at 39 °C instead of 37 °C accelerates the inhibition [48].

## 4. Correlation as a Measure of Method Reliability

The similarity of the average half-lives in the majority of the studies may have led to the belief that the methods are consistent. However, more recent studies focused on the correlation between the mRNA half-lives, which turned out to be surprisingly low [10,11,12]. This means that different methods classify different mRNAs as stable or unstable.

There are two types of correlation coefficients: the Pearson’s product-moment and Spearman’s rank correlation coefficients. When the distribution of the half-lives is not Gaussian then the Spearman’s rank correlation should be used. The rank correlation coefficient is less sensitive to outliers. The higher the correlation between two methods, the more likely that the same mRNAs will be classified as stable or unstable.

There is no uniform way to delimit the lowest acceptable value of correlation. Generally, values between 0.3 and 0.5, and between 0.5 and 0.7 reflect low and moderate correlations, respectively. Values above 0.7 are considered high. It is instructive to consider a field in molecular biology where correlation is used to assess the robustness of predictions. For example, correlations have been used to predict biological age of an individual from unknown tissue samples based on epigenetic markers. Methylation of CG dinucleotides (CpG), a classical epigenetic mark, increases with age. Accordingly, the CpG methylation of appropriately selected promoters can be used to predict age based on biological samples. The correlation between the CpG methylation percentage and age can be then used for linear regression to predict age from biological samples [49,50]. Different gene selections yield different values for the correlations and thus have different predictive power. Some selections yield correlation coefficients of 0.7, while others surpass even 0.9, which is equivalent to a prediction error of ±3 years in the range above 20 years of age [49,50]. Thus, as rule of thumb, a correlation coefficient of 0.7 or more indicates that the respective methods can be used to determine RNA half-lives with a relatively high precision.

## 5. Internal Consistency and Inter-Method Reliability: From Simplicity to Perplexity

In one of the first comparative studies, the half-lives of 11 mRNAs were measured using the temperature sensitive polymerase (*rpb1-1*) and in vivo labelling with [^32^P]-phosphate [3]. The two methods yielded similar decay rate constants for around half of the examined RNA species but four mRNAs displayed marked discrepancies. By analyzing the poly(A)+ mRNA, the authors suggested that the kinetics of deadenylation may account for the discrepancy.

This study was followed by the first genome-wide comparison [10], which examined the consistency of the *rpb1-1* method. The internal consistency of a specific method can be assessed by calculating the correlation between two studies using the same method variant. The authors found that the correlation was nearly three times higher for highly expressed genes (*R* = 0.48 at the 90th percentile) than for weakly expressed genes (*R* = 0.15 at the 10th percentile), which suggests that the weak signal intensity may introduce considerable measurement error.

A more recent study assessed both internal consistency and inter-method reliability. Two variants of the metabolic labelling employing different pulsing protocols yielded uncorrelated half-lives [11]. The comparison of five different studies using the same variant of the same method (*rpb1-1* allele of the polymerase) indicated a low reproducibility of the method, with the Spearman’s rank correlation coefficient between five different studies ranging between 0.28 and 0.77 [11]. Furthermore, there was no correlation between the transcriptional inhibition and metabolic labelling studies.

To explain the discrepancy between the transcriptional inhibition and in vivo labelling, the study using a variant of the metabolic labelling, the cDTA, found that the *rpb1-1* allele increases mRNA stability even at permissive temperature [51]. Similar stabilization was found with gene control measurements when decay was measured in cells with mutant polymerases with reduced elongation rates [52]. Thus, there is a global interdependence between the transcription and degradation machinery [12,53]. This includes both direct and indirect effects. Transcriptional inhibition reduces Xrn1p levels directly, resulting in diminished RNA decay rates. An indirect effect on RNA synthesis rates has been also suggested, mediated by a specific transcriptional repressor [51].

Since two studies using metabolic labelling did not correlate with each other and with the inhibition studies [11], it was difficult to justify why metabolic labelling should supersede the transcriptional inhibition methods. This conundrum was resolved in part by a recent study using a multiplexed version of the gene control method using the TET system, which yielded half-lives with a high correlation to one version of the metabolic labelling, the cDTA, with a rank correlation coefficient of 0.77 [12]. This high correlation can be particularly appreciated from the perspective that the two studies are methodologically independent, since they introduce different perturbations into the cells. Despite the large positive correlation between the gene control and the cDTA variant of metabolic labelling, there is a significant difference in the median half-lives. The five-times-longer half-life in the cDTA study may be explained by the slow incorporation of the labelled nucleotides into the RNA. The slow incorporation may in turn caused by the slow cellular uptake of uracil, which is controlled by a complex regulatory circuit. The uracil inhibits its own import by the uracil permease Fur4p. The half-lives of both the RNA and protein products of the *FUR4* gene are reduced upon exposure to uracil [54]. Thus, the high concentration pulse of labelled nucleotides may slow down the uptake and incorporation of labelled uracil.

It is somewhat surprising that there is no correlation between two variants of the metabolic labelling [11]. What may explain this discrepancy? This inconsistency cannot be explained unequivocally because the applied protocols differ at multiple points. The study by Munchel et al. employs the pulse–chase approach, the RNA is labelled with low nucleotide concentration (0.2 mM TU for 3 h in a log-phase culture) and the RNA is quantified by high-throughput sequencing. On the other hand, cDTA uses the approach to equilibrium method upon exposing the cells to high nucleotide concentrations (5 mM TU for 6 min in a log-phase culture), and the RNA was quantified by microarray. A comparison of multiple in vivo labeling studies to an independent method, the multiplexed gene control, can help to narrow down the reasons for the discrepancy. The multiplexed gene control method correlates more with the cDTA than with the RATE-seq [12]; and RATE-seq employs RNA-seq for RNA quantification [55]. Thus, microarray may have delivered more precise measurements than RNA-seq. This conclusion is strengthened by knowing that the RATE-seq was expected to yield more robust half-life estimates because multiple time points were measured in RATE-seq, whereas only a single time point was measured in the cDTA study.

The methods applied to measure the half-life can perturb the cell or RNA turnover, and each method can have a differential effect on the stability of individual mRNAs. Therefore, it is possible that a sequence motif identified with a particular method reflects the interaction of the perturbation with the cell rather than the original half-life. Thus, using two independent methods or cross-validated methods can ensure that a motif identified reflects the parameter to be measured. This is particularly important because RNA decay may be determined not only by simple sequence motifs but also by RNA secondary structure [56] and by the translation efficiency of the mRNA [57].

## 6. Comparison of Half-Lives in Mammalian Cells

A systematic comparison of mRNA stabilities in mammalian cells is hampered by the large variety of cell lines and differentiation states. A study using global transcriptional inhibition with actinomycin D reported a median mRNA half-life of 10 h [58]. Two studies used 4-thiouridine (4sU) labeling in the same cell line (NIH3T3) [14,59]; the correlation between these two data sets was moderate (*R* = 0.64), having median half-lives of 7.6 and 4.6 h. A metabolic labelling study with 4sU labelling reported considerably shorter mRNA half-lives in dendritic cells, in the range between 10 and 70 min [47]. Thus, cross-validation of methods will be important also for mammalian cells.

## 7. Identification of the RNA Degradation Machinery: Xrn1, Exosome and the Nonsense Mediated Decay

Next, we will review how the major components of the mRNA degradation machinery were discovered, from a historical-methodological perspective. All the major components, the Xrn1 and exosome, and the major surveillance pathway (nonsense mediated decay) were identified in the yeast *Saccharomyces cerevisiae*.

### 7.1. Xrn1: 5′-to-3′ Degradation

The Xrn1 protein was identified biochemically as a 5′-to-3′ ribonuclease activity on uncapped mRNA. Capped mRNAs were shown to be quite resistant to degradation by the purified Xrn1p [60]. The 5′ cap, found on the 5′ end of the messenger RNAs, consists of a methylated guanine nucleotide connected to the mRNA via an unusual triphosphate linkage. Upon identifying the gene encoding the Xrn1p, it was shown to be a non-essential gene but its deletion causes a marked growth defect [61]. To link the in vivo activity of Xrn1 to mRNA decay, a transcriptional inhibition study using thiolutin and phenanthroline revealed that Xrn1 affects particularly the stability of mRNAs with short lives [62]. Subsequently, a gene control experiment with the GAL promoter confirmed the involvement of *XRN1* in the regulation of the half-life of the unstable *MFA2* and stable *PGK1* mRNAs [63]. The methods relying on *rpb1-1* was used to show that the glucose-dependent stability of an mRNA is Xrn1-dependent [64]. Xrn1 also controls the levels of sense-antisense mRNA pairs, which can arise as a result of convergent gene expression [65].

Little is known about the regulation of Xrn1; subcellular sequestration of Xrn1 may play an important role. The protein is diffusely distributed in the cells in exponentially growing cultures but becomes localized to the cytoplasmic P-bodies at the diauxic shift. Subsequently, at the post-diauxic stage, Xrn1 is localized to the plasma membrane, which prevents mRNA degradation, as shown by the reduced decay of the MFα2 mRNA using a TET-based gene control method [66].

It has been shown that the Xrn1p undergoes rapid evolution, as evidenced by the large sequence divergence of *XRN1* among related yeast species [67]. This may in part explain the multiple functions of Xrn1p. Xrn1p is primarily an exoribonuclease but has other functions in the cell, which are often denoted by different aliases. Of practical importance is the *KEM1* alias, which refers to Kar-enhancing mutations: haploid cells with *xrn1* deletion have a very low mating efficiency due to a defective karyogamy [68]. The defective fusion of two nuclei makes the construction of *xrn1* null diploid cells difficult.

### 7.2. Exosome: 3′-to-5′ Degradation

The main component of the second pathway of mRNA decay is the exosome, which is a multi-protein complex with a 3′-to-5′ ribonuclease activity [69]. The exosome has also a nuclear function. It was isolated and identified by mass-spectrometry [70]. It is more difficult to study the effect of the exosome because the deletion of its components is lethal. It is however possible to delete the *SKI* genes; the Ski proteins bridge the exosome and the RNA. The in vivo activity of the exosome was studied by combining the inactivation of the decapping protein Dcp1 with the deletion of *SKI8* [71]. Individual mutation of each of the genes had little effect on the decay rates of the *GAL* and *MFA2* RNAs as studied by the gene control method, using the *GAL* promoter. However, when both of these proteins were inactivated, the half-life of the mRNAs lengthened around 6–7 times [71].

### 7.3. Nonsense Mediated Decay (NMD)

So far, few stability sequence motifs have been identified with dominant effect on stability when transferred to other mRNAs. A simple sequence that consistently changes the half-life of the mRNAs is the premature stop codon [72,73,74]. Just like the main components of the eukaryotic mRNA degradation machinery, this phenomenon was discovered in budding yeast. Using radioactive labelling, it was shown that premature stop codons—in a position dependent manner—alter the stability of mRNAs without affecting the transcription rate [73]. This phenomenon, known as nonsense mediated decay (NMD), was shown to be mediated by the Upf1p, using transcriptional inhibition with the *rpb1-1* mutant [72]. The closer the stop codon to the 5′-end of the stable *PGK1* mRNAs was, the more strongly the mRNA got destabilized [74]. NMD was also confirmed with gene control experiments [45]. There are two main sources of mRNAs subject to NMD. First, premature stop codons can arise by mutations during transcription, a phenomenon termed transcriptional infidelity [75]. Second, a fraction of the unspliced RNA escapes from the nucleus into the cytoplasm and the retained introns contain a large number of premature stop codons [45,46]. NMD can also affect mRNAs that lack premature stop codons but are prone to out-of-frame translation [76].

## 8. Conclusions

In vivo labelling and inhibition of RNA expression have been used for at least four decades to measure RNA half-lives. Surprisingly, only recent studies unveiled that the two methods yield half-lives with no or minimal correlation. A more recent study showed that there is a high correlation between specific variants of the in vivo metabolic labelling and gene control methods. The consistency between two independent methods indicates high inter-method reliability. Thus, some variants of the genetic control and metabolic labelling may reflect the half-lives the most faithfully. However, there is no guarantee that using a method class, let it be metabolic labelling or gene control, will deliver the half-lives faithfully. If the conditions are not optimal, such as the concentration of the labelled nucleotide or the expression level of the activator that controls the gene, the measured half-lives and RNA synthesis rates may be inconsistent.

The most robust methods are expected to have played a major role in the identification of the molecular machinery of mRNA degradation. In most cases, in vitro biochemical studies were first undertaken. Subsequently, the in vivo activities of the identified components were confirmed by most of the major methods. Thus, all methods capture RNA decay, at least qualitatively. However, current evidence indicates that specific variants of the gene control and metabolic labelling methods can be used with high reliability to quantify the differential stability of mature mRNAs. We expect that the confirmation of mRNA stabilities by two different methodologies and further developments in the analysis of inter-method reliability will help to identify mRNA stability determinants.
